# Paramyxoviruses respiratory syncytial virus, parainfluenza virus, and human metapneumovirus infection in pediatric hospitalized patients and climate correlation in a subtropical region of southern China: a 7-year survey

**DOI:** 10.1007/s10096-019-03693-x

**Published:** 2019-09-05

**Authors:** Wen-Kuan Liu, De-Hui Chen, Wei-Ping Tan, Shu-Yan Qiu, Duo Xu, Li Zhang, Shu-Jun Gu, Rong Zhou, Qian Liu

**Affiliations:** 1 State Key Laboratory of Respiratory Diseases, National Clinical Research Center for Respiratory Disease, The First Affiliated Hospital of Guangzhou Medical University, Guangzhou Institute of Respiratory Health, Guangzhou Medical University, Guangzhou, China; 2 Department of Pediatrics, The First Affiliated Hospital of Guangzhou Medical University, Guangzhou, China; 3 Department of Pediatrics, Sun Yat-Sen Memorial Hospital, Sun Yat-Sen University, Guangzhou, China; 4 Scientific Research Center, The First Affiliated Hospital of Guangdong Pharmaceutical University, Guangzhou, China; 5 Department of Pediatrics, Dongguan Eighth People’s Hospital, Dongguan, China

**Keywords:** Respiratory syncytial virus, Parainfluenza virus, Human metapneumovirus, Acute respiratory illness, Epidemiology, Meteorological conditions

## Abstract

To investigate the features of paramyxovirus respiratory syncytial virus (RSV), parainfluenza virus (PIV), and human metapneumovirus **(**HMPV) infection and determine the effect of meteorological conditions in Guangzhou, a subtropical region of southern China. We collected 11,398 respiratory samples from hospitalized pediatric patients with acute respiratory illness between July 2009 and June 2016 in Guangzhou. The samples were tested simultaneously for 18 respiratory pathogens using real-time PCR. Local meteorological data were also collected for correlation analysis. Of 11,398 patients tested, 5606 (49.2%) patients tested positive for one or more pathogens; RSV, PIV, and HMPV were the first, sixth, and ninth most frequently detected pathogens, in 1690 (14.8%), 502 (4.4%), and 321 (2.8%) patients, respectively. A total 17.9% (4605/5606) of patients with positive results had coinfection with other pathogens. Significant differences were found in the prevalence of RSV, PIV, and HMPV among all age groups (*p* < 0.001). RSV and HMPV had similar seasonal patterns, with two prevalence peaks every year. PIV appeared alternatively with RSV and HMPV. Multiple linear regression models were established for RSV, PIV, and HMPV prevalence and meteorological factors (*p* < 0.05). RSV and PIV incidence was negatively correlated with monthly mean relative humidity; RSV and HMPV incidence was negatively correlated with sunshine duration; PIV incidence was positively correlated with mean temperature. We described the features of paramyxovirus infection in a subtropical region of China and highlighted the correlation with meteorological factors. These findings will assist public health authorities and clinicians in improving strategies for controlling paramyxovirus infection.

## Introduction

Respiratory syncytial virus (RSV), parainfluenza virus (PIV), and human metapneumovirus **(**HMPV) are enveloped, nonsegmented, negative-sense, single-stranded RNA viruses belonging to the *Paramyxoviridae* family*.* These viruses are a significant cause of morbidity and mortality globally, especially among children in developing countries [1–7]. RSV is the most important pathogenic infection of childhood worldwide, causing a variety of manifestations from mild upper respiratory tract illnesses or otitis media to severe and potentially life-threatening lower respiratory tract illnesses [6, 8–11]. Four PIV types (PIV1–4) have been identified [12, 13]. PIV1 and PIV2 are best known as the cause of croup whereas PIV3 is a common cause of bronchiolitis and pneumonia [7]. PIV4 infection has low prevalence [13]. HMPV was first discovered in patients with acute respiratory illness (ARI) in 2001 [14]. Since then, HMPV has been associated with ARI in children as well as elderly and immunocompromised adults [15–17]. RSV, PIV, and HMPV are also important causes of nosocomial infection, which might be life-threatening in certain individuals, such as transplant or immunocompromised patients [18–25]. Until now, no effective vaccine for RSV has been available. The RSV-specific monoclonal antibody palivizumab has been advocated for use as prophylaxis in high-risk patients against RSV infection [26–28]. However, there is no available vaccine or specific antiviral treatment for PIV and HMPV infection. Consequently, it is imperative to conduct further research, especially in low- and middle-income countries, to understand the epidemiological features of these pathogens in different areas and populations.

In general, the prevalence of viruses can vary because of factors such as geographical location, climatic conditions, population, and social activity [29]. Guangzhou, which is located on the subtropical coast of China, has a maritime subtropical monsoon climate. Guangzhou is China’s first gateway hub to Southeast Asia and Oceania. The city is densely populated and frequent exchanges of domestic and international personnel and materials take place in the area. Guangzhou has been a hotbed of activity for various respiratory pathogens. Investigation of respiratory pathogen epidemics in the region is critical.

In this study, we analyzed paramyxovirus infection among children hospitalized with ARI over a 7-year period in Guangzhou, and we collected local meteorological data for climate correlation analysis. These data will be helpful for the prevention and control of these viruses.

## Materials and methods

### Study design and respiratory samples, and meteorological data collection

We performed a cross-sectional study in three tertiary hospitals between July 2009 and June 2016 in Guangzhou, southern China. Pediatric patients (*n* = 11,398) hospitalized with ARI were enrolled in this study. The detail inclusion criteria were pediatric patients (≤ 14 years old) who presented with at least two of the following symptoms: cough, pharyngeal discomfort, nasal obstruction, rhinitis, or dyspnea during the previous week. Patients who were diagnosed with pneumonia by chest radiography during the previous week were also included in the study, even if they did not show the clinical features described above. Some patients who had been cured and discharged some time ago but were then readmitted because of a new episode of ARI were included as new cases if met the recruitment criteria; otherwise, they were excluded. Chest radiography was conducted according to the clinical situation of the patient. Respiratory samples, including throat swab, sputum, or bronchoalveolar lavage fluid, were collected from the enrolled patients for routine screening of respiratory viruses, *Mycoplasma pneumoniae* (MP), and *Chlamydophila pneumoniae* (CP), according to established clinical protocols [13]. The samples were refrigerated at 2–8 °C in viral transport medium, transported on ice, and analyzed immediately or stored at − 80 °C before analysis, as described previously [30].

We also collected meteorological data of Guangzhou (longitude E112° 57′ to E114° 3′, latitude N22° 26′ to N23° 56′), including the monthly mean temperature (°C), mean relative humidity (%), rainfall (mm), sunshine duration (h), mean wind speed (m/s), mean air pressure (hPa), and mean vapor pressure (hPa) from the China Meteorological Administration between July 2009 and June 2016.

### Real-time PCR for detection of RSV, PIV, HMPV, and common respiratory pathogens

TaqMan real-time PCR was conducted to detect RSV, PIV1–4, HMPV, and other 12 common respiratory pathogens, including influenza A virus (infA), influenza B virus (infB), human rhinovirus (HRV), enterovirus (EV), four types of coronaviruses (HCoV-229E, -OC43, -NL63, and -HKU1), adenovirus (ADV), human bocavirus (HBoV), MP, and CP, as previously reported [13]. Briefly, real-time-PCR and RNA/DNA extraction kits were purchased from Guangzhou HuYanSuo Medical Technology Co., Ltd. RNA/DNA was extracted from 200-μL samples, according to the manufacturer’s protocol. The cycling conditions were 48 °C for 10 min, 94 °C for 2 min, and then 40 cycles of 94 °C for 10 s and 55 °C for 35 s. The amplified products were detected using the Applied Biosystems 7500 Real-Time PCR System (Life Technologies, Singapore). The sensitivity of the detection kits was 500 copies/mL and 1000 copies/mL for the target DNA and RNA, respectively.

### Statistical analysis

Statistical analysis was performed using SPSS 19.0 (SPSS Inc., Chicago, IL, USA). Numerical data were presented as mean ± standard deviation for continuous variables of meteorological data, percentage for normal discrete variables, or median (interquartile range, IQR) for age distribution. Categorical data were compared with the chi-squared test. Multiple linear regression analysis was performed with RSV, PIV, and HMPV prevalence as dependent variables and meteorological factors as independent variables. Linear correlations of meteorological independent variables were analyzed to exclude any effect on the final multiple linear regression analysis. The independent variable mean temperature drop 1 month (mean temperature in the preceding month) was also included as an independent variable in the multiple linear regression analysis because of its delay effect. A *p* value < 0.05 (two-tailed) was considered statistically significant.

## Results

### Patients and paramyxovirus infection

Over a 7-year period, a total of 11,398 patients were enrolled in the study and screened for RSV, PIV, HMPV, and 12 respiratory pathogens (Table 1). The median age of patients was 1.8 years (IQR, 0.8–3.8), and the sex ratio was 1.8:1. Of the 11,398 patients tested, 5606 (49.2%) had positive results for one or more of the pathogens of interest, and 17.9% (1001/5606) of positive patients were found to have coinfection with two or more pathogens. The median age of pathogen-positive patients was 1.5 years (IQR, 0.7–3.0), and positive patients had a higher sex ratio (1.9:1) than patients who tested negative for all pathogens (1.7:1) (*p* = 0.002).

**Table 1:** Respiratory pathogens detected among hospitalized pediatric patients with acute respiratory illness: Guangzhou, Southern China

Pathogens	No. of positive samples with potential pathogens															Prevalence, % (n = 11,398)
	RSV	PIV	HMPV	infA	infB	HRV^a^	EV	229E	OC43	NL63	HKU1	ADV	HBoV	MP	CP	
RSV	1690	38	16	95	25	45	73	10	29	10	3	38	29	38	8	14.8
PIV		502	7	25	8	18	29	5	28	2	1	17	18	36	1	4.4
HMPV			321	12	5	3	10	3	14	1	1	9	7	9	0	2.8
infA				839	34	8	41	7	38	4	4	23	13	46	1	7.4
infB					300	6	9	0	9	0	1	4	4	15	2	2.6
HRV^a^						402	16	2	11	1	1	14	17	21	6	5
EV							498	4	10	6	1	24	15	21	5	4.4
229E								64	14	2	0	3	0	3	0	0.6
OC43									346	2	1	14	9	27	3	3.0
NL63										60	1	5	3	3	1	0.5
HKU1											38	2	1	3	1	0.3
ADV												621	14	36	0	5.4
HBoV													248	14	0	2.2
MP														760	2	6.7
CP															77	0.7
Single pathogen	1314	318	243	546	203	261	286	26	185	28	19	458	136	531	51	40.4
Co-pathogens	376	184	78	293	97	141	212	38	161	32	19	163	112	229	26	8.8

^a^HRV detected since January 2012, and a total of 8084 cases were collected. *229E*, human coronavirus 229E; *OC43*, human coronavirus OC43; *NL63*, human coronavirus NL63; *HKU1*, human coronavirus HKU1; *MP*, *Mycoplasma pneumoniae*; *CP*, *Chlamydophila pneumoniae*

**Fig. 1:**
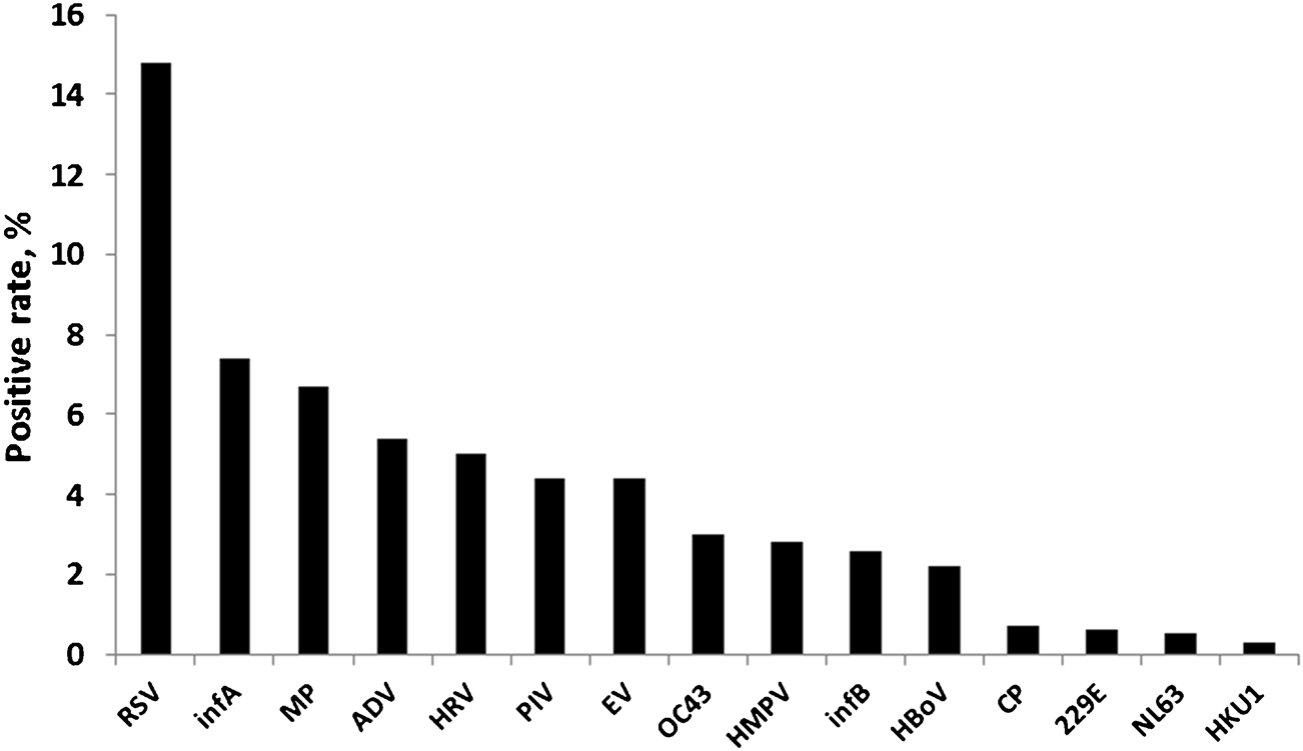
Respiratory pathogen distribution among hospitalized pediatric patients with acute respiratory illness in Guangzhou, China

RSV, PIV, and HMPV were the first, sixth, and ninth most frequently detected pathogens, with prevalence of 14.8% (1690/11398), 4.4% (502/11398), and 2.8% (321/11398), respectively (Fig. 1, Table 1). The median age of patients who tested positive for these paramyxoviruses was 1.3 years (IQR, 0.4–1.8), 1.7 years (IQR, 0.6–2.5), and 2.1 years (IQR, 0.8–3.0), respectively. The sex ratio of patients positive for these three viruses was 2.4:1, 2.4:1, and 2:1, respectively.

### Age distribution of patients with RSV, PIV, or HMPV infection

**Fig. 2:**
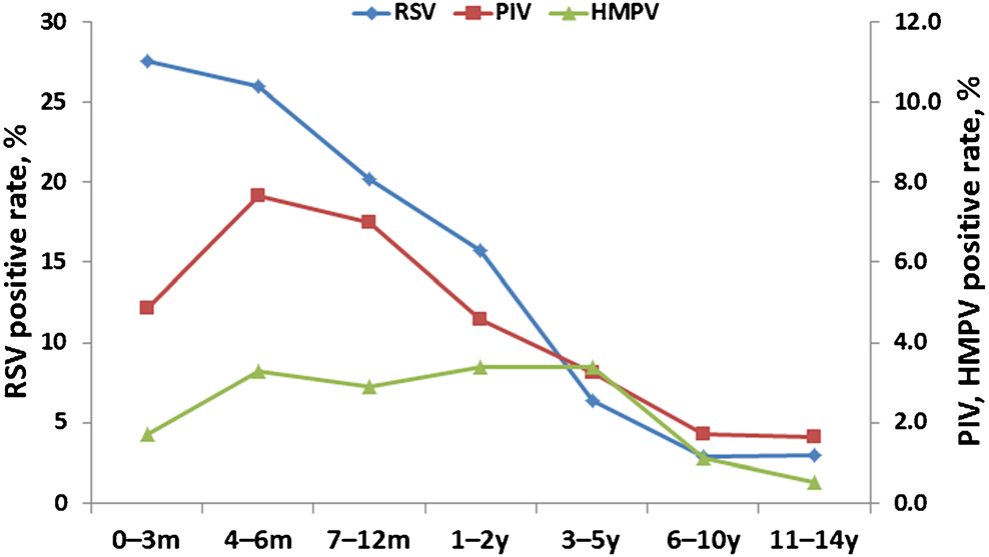
Age distribution of RSV, PIV, and HMPV among hospitalized patients with acute respiratory illness, Guangzhou

Patients were divided into seven age groups to clarify the age distributions for these three paramyxoviruses, as follows: age 0–3 months, 4–6 months, 7–12 months, 1–2 years, 3–5 years, 6–10 years, and 11–14 years. Significant differences were found in the prevalence of RSV, PIV, and HMPV among all age groups (*p* < 0.001), and prevalence declined with age for RSV. Peak prevalence was found in patients aged 4–6 months (7.7%, 83/1084) for PIV; high HMPV prevalence was found in patients 4 months to 5 years old, 2.9% (35/1203) to 3.4% (124/3601) (Fig. 2).

### Seasonal distribution of RSV, PIV, and HMPV

**Fig. 3:**
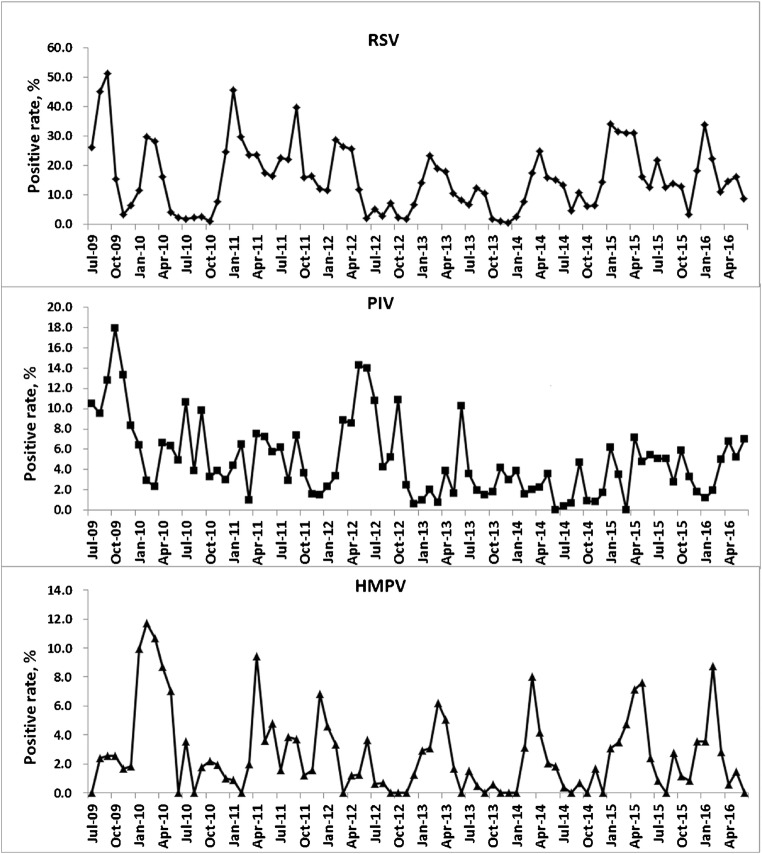
Seasonal distribution of RSV, PIV, HMPV in hospitalized pediatric patients with acute respiratory illness, Guangzhou

Overall, in the 7-year period study, RSV and HMPV had similar seasonal patterns as well as two clear prevalence peaks each year. The larger peak of RSV and HMPV prevalence appeared during the change of season from winter to spring, mainly occurring in February to April every year. The smaller peak was mainly observed in August to October each year, during the shift from summer to autumn (Fig. 3). PIV prevalence increased as autumn turned to winter and summer turned to autumn, and appeared between peaks of RSV and HMPV prevalence (Fig. 3).

### Correlation of RSV, PIV, and HMPV epidemics and meteorological conditions

To explore the correlation of paramyxovirus prevalence with climate conditions in Guangzhou, we collected meteorological data for the 7-year period. Between July 2009 and June 2016, the mean temperature was 21.8 ± 5.8 °C, mean relative humidity was 77.2 ± 7.3%, sunshine duration was 132.7 ± 59.5 h, mean wind speed was 2.2 ± 0.6 m/s, rainfall was 175.2 ± 165.9 mm, mean air pressure was 1005.6 ± 6.0 hPa, and mean vapor pressure was 21.3 ± 7.4 hPa.

Multiple linear regression analysis was conducted to explore the correlation between meteorological conditions and paramyxovirus prevalence. We first analyzed linear correlations among meteorological independent variables. We excluded the independent variables mean air pressure (adjusted *R*^2^ = 0.793, *p* < 0.001) and mean vapor pressure (adjusted *R*^2^ = 0.929, *p* < 0.001), which were linearly associated with mean temperature, and we excluded rainfall (adjusted *R*^2^ = 0.278, *p* < 0.001), which was strongly correlated with mean relative humidity. Thus, the independent variables included in the final multiple linear regression analysis were mean temperature, mean relative humidity, sunshine duration, and mean wind speed. Multiple linear regression models were established for RSV, PIV, and HMPV prevalence and meteorological data (*p* < 0.05) (Table 2).

**Table 2:** Multiple linear regression analysis of correlation between RSV, PIV, HMPV epidemics and meteorological factors, Guangzhou

Pathogen	Model summary		Correlation coefficients		
	Model significance (ANOVA)	Adjusted *R*^2^	Meteorological factor	Standard coefficient	*p* value
RSV	*p* = 0.048	0.069	Mean temperature (°C)	0.59	0.14
			Mean relative humidity (%)	*− 0.573*	*0.034*
			Mean wind speed (m/s)	4.005	0.144
			Sunshine duration (h)	*− 0.097*	*0.007*
PIV	*p* < 0.001	0.197	Mean temperature (°C)	*0.328*	*0.009*
			Mean relative humidity (%)	*− 0.194*	*0.02*
			Mean wind speed (m/s)	**−** 0.699	0.404
			Sunshine duration (h)	**−** 0.2	0.246
HMPV	*p* < 0.001	0.188	Mean temperature (°C)	**−** 0.016	0.863
			Mean relative humidity (%)	**−** 0.057	0.357
			Mean wind speed (m/s)	**−** 0.81	0.2
			Sunshine duration (h)	*− 0.024*	*0.004*

Multiple linear regression analysis was performed for monthly prevalence of three paramyxoviruses as the dependent variable, and monthly mean temperature, mean relative humidity, sunshine duration, and mean wind speed as the independent variables

Data in italics are significant

**Fig. 4:**
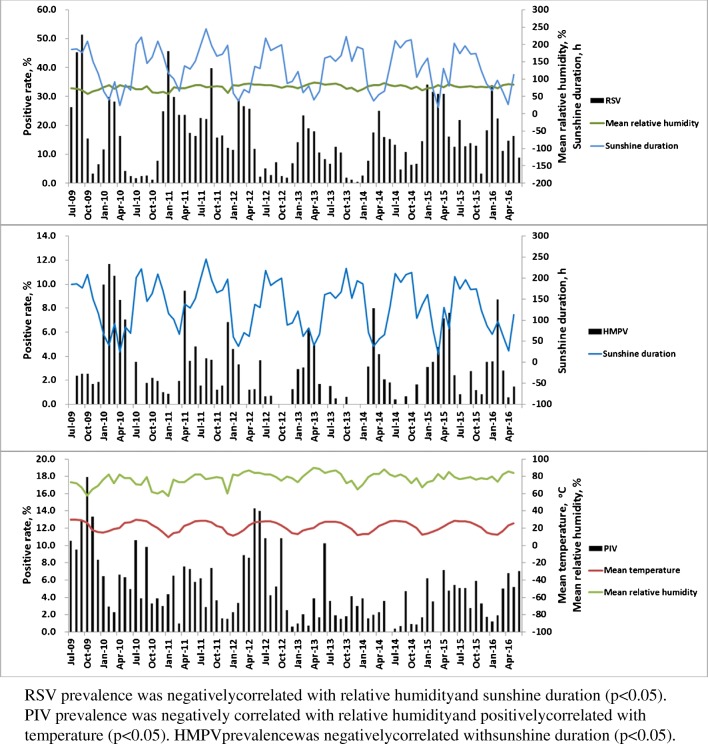
Correlation of RSV, PIV, and HMPV prevalence with meteorological conditions in Guangzhou, China.

RSV prevalence was negatively correlated with relative humidity and sunshine duration (coefficient = − 0.573 and − 0.097, respectively) (*p* < 0.05). PIV prevalence was negatively correlated with relative humidity (coefficient = − 0.194) and positively correlated with temperature (coefficient = 0.328) (*p* < 0.05). HMPV prevalence was negatively correlated with sunshine duration (coefficient = − 0.024) (*p* < 0.05) (Table 2, Fig. 4). Mean temperature drop 1 month (mean temperature in the preceding month) was also included as an independent variable in the analysis; however, no effective regression model was established (*p* > 0.05).

## Discussion

ARI is one of the most common human diseases resulting in high mortality and mobility, and it is predominantly caused by respiratory viruses [31, 32]. Paramyxoviruses, including RSV, PIV, and HMPV, are the most important respiratory viruses in patients with ARI all over the world, especially among children under 5 years old [33, 34]. These viruses are also an important cause of nosocomial infection [18–25]. Currently, no effective vaccines or drugs have been developed against these viruses, except palivizumab for RSV prophylaxis [18]. Thus, additional research must be carried out. In the present study, we sought to analyze the features of paramyxovirus infection and correlation with meteorological conditions in a subtropical region of southern China, by collecting respiratory samples from pediatric patients (≤ 14 years old) hospitalized with ARI in Guangzhou and testing for RSV, PIV, HMPV, and other common respiratory pathogens. Meteorological data were also collected between July 2009 and June 2016, for further correlation analysis. The goal of the study was to reveal information that could be useful in the prevention and control of these viruses.

The median age of the 11,398 enrolled pediatric patients was 1.8 years (IQR, 0.8–3.8), indicating the high public health burden among infants and young children, as previously reported [33, 34]. Nearly half of all patients had positive test results for one or more of the pathogens of interest; moreover, all pathogens in the panel were detected, indicating the complexity and diversity of ARI etiology (Table 1). Higher pathogen prevalence was found among male patients than female ones (*p* = 0.002), similar to previous reports [35]. RSV, infA, MP, ADV, HRV, and PIV were the six most frequently detected pathogens in this study (Fig. 1); these results are consistent with previous reports from China as well as international studies [1, 2, 35, 36].

RSV, PIV, and HMPV are the most important respiratory viruses, causing lower respiratory illnesses among children worldwide [4–6, 33]. In this study, RSV, PIV, and HMPV were the first (14.8%), sixth (4.4%), and ninth (2.8%) most frequently detected pathogens (Table 1, Fig. 1). In children with positive results for these three paramyxoviruses, the median age was highest in children who were HMPV positive and lowest for those positive for RSV. RSV mostly affected children under 2 years old, and the prevalence decreased with age (*p* < 0.001). PIV showed peak prevalence among patients aged 4–6 months (*p* < 0.001), and there was high HMPV prevalence among patients aged 4 months to 5 years (*p* < 0.001) (Fig. 2). The different age distributions of these three viruses may be helpful in determining appropriate pediatric care and disease diagnosis; however, laboratory testing is still necessary because of the complex diversity and similarities in clinical manifestations of respiratory pathogens [1, 2, 4].

In general, the epidemiology of the main respiratory viruses in patients with ARI has been closely monitored in developed countries; however, these data are less available in developing countries, mostly because of the high cost of these studies. RSV is known to occur in well-defined, recurrent epidemics during the cold season in temperate climates [6], with peaks occurring more often during the rainy season in tropical and subtropical areas; locations close to the equator have less consistent patterns, with some showing nearly continuous RSV activity and varying seasonal peaks [37]. In this study, we found that RSV occurred during the change of seasons from winter to spring and from summer to autumn. This pattern is similar to those in previous reports [38]. HMPV had a similar epidemic pattern to RSV, consistent with previous reports from other subtropical areas [39, 40], and differed from the pattern in temperate climates, which peaks at the end of winter or in early spring [41, 42]. In previous reports from the USA, PIV is second only to RSV as a cause of hospitalization for ARI (2–17%) among children aged younger than 5 years [12, 43, 44]. Seasonal peaks of PIV are mostly driven by PIV-3 and PIV-1, whereas there are a small number of PIV2 and especially PIV4 infections [45, 46]. In this study, PIV was isolated throughout the year and appeared to alternate with peaks in RSV and HMPV infection, increasing as autumn turned to winter and summer turned to autumn (Fig. 3). These results differ from previous reports of biennial PIV epidemics [45, 47]. The different geographic location might lead to the different seasonal distribution of PIV observed in the present study.

In addition to pathogenic characteristics, pathogenic epidemics are closely related to geographic environment, local climate, social development level, population structure, ethnic characteristics, social interaction, and so forth. Guangzhou has a maritime subtropical monsoon climate, with high temperatures (mean temperature 21.8 ± 5.8 °C) and high relative humidity (77.2 ± 7.3%). Investigation of respiratory pathogen epidemics in the region is of great importance. In this study, we analyzed the correlation between the prevalence of paramyxoviruses among pediatric hospitalized patients and meteorological conditions in Guangzhou. Multiple linear regression analysis was performed with monthly RSV, PIV, and HMPV prevalence as the dependent variable and current mean temperature (or mean temperature in the preceding month), mean relative humidity, mean wind speed, and sunshine duration as the independent variables. Regression models were established for RSV, PIV, and HMPV using the current monthly temperature model (*p* < 0.05) (Table 2). However, regression models using mean temperature in the preceding month model were not established. In general, the trend of associations between climate factors and respiratory pathogen activity varies with geographic location [35, 48–53]. In this study, RSV and HMPV had similar seasonal distribution patterns, and both were negatively correlated with sunshine duration (Table 2, Fig. 4); this might be owing to the sensitivity of RSV and HMPV to ultraviolet light. In subtropical and temperate regions, RSV prevalence is more consistently positively correlated with lower temperatures and higher relative humidity [48]. However, we found a negative correlation between RSV prevalence and relative humidity (*p* < 0.05) (Table 2, Fig. 4), which might be owing to the high relative humidity in the Guangzhou region (monthly mean relative humidity 77.2 ± 7.3%). PIV had an alternating seasonal distribution pattern with RSV and HMPV in this study. PIV incidence was negatively correlated with relative humidity, similar to RSV; however, PIV incidence was positively correlated with mean temperature, and the absolute value of the correlation coefficient of relative humidity (|− 0.194|) was smaller than the absolute value of the correlation coefficient of temperature (|0.328|) (Table 2), which means that the effect of temperature on the distribution of PIV was greater than the relative humidity. This might explain the different distribution patterns between RSV and PIV. Overall, the established models were found to be of value for understanding the epidemic patterns of RSV, PIV, and HMPV (Fig. 4).

Some limitations of this study should be noted. First, because our study was mainly focused on the circulation of paramyxoviruses among hospitalized patients with ARI, paramyxoviruses in outpatients and the asymptomatic population were not included. Second, many factors can affect virus epidemics; meteorological data analysis alone may be insufficient to reach a final conclusive interpretation. Third, the current study was only conducted in three hospitals and may not be representative of the overall population.

In conclusion, this study provided a better understanding of paramyxoviruses infection and highlighted the correlation with climate factors, revealing the potential for modeling and risk assessment. The findings of this work will help public health authorities and clinicians to improve strategies for controlling paramyxoviruses infection.
